# Predicting circRNA–Disease Associations Based on Improved Collaboration Filtering Recommendation System With Multiple Data

**DOI:** 10.3389/fgene.2019.00897

**Published:** 2019-09-25

**Authors:** Xiujuan Lei, Zengqiang Fang, Ling Guo

**Affiliations:** ^1^School of Computer Science, Shaanxi Normal University, Xi’an, China; ^2^College of Life Sciences, Shaanxi Normal University, Xi’an, China

**Keywords:** circRNA–disease association, collaboration filtering, multiple biological data, recommendation system, neighbor information

## Abstract

With the development of high-throughput techniques, various biological molecules are discovered, which includes the circular RNAs (circRNAs). Circular RNA is a novel endogenous noncoding RNA that plays significant roles in regulating gene expression, moderating the microRNAs transcription as sponges, diagnosing diseases, and so on. Based on the circRNA particular molecular structures that are closed-loop structures with neither 5′-3′ polarities nor polyadenylated tails, circRNAs are more stable and conservative than the normal linear coding or noncoding RNAs, which makes circRNAs a biomarker of various diseases. Although some conventional experiments are used to identify the associations between circRNAs and diseases, almost the techniques and experiments are time-consuming and expensive. In this study, we propose a collaboration filtering recommendation system–based computational method, which handles the “cold start” problem to predict the potential circRNA–disease associations, which is named ICFCDA. All the known circRNA–disease associations data are downloaded from circR2Disease database (http://bioinfo.snnu.edu.cn/CircR2Disease/). Based on these data, multiple data are extracted from different databases to calculate the circRNA similarity networks and the disease similarity networks. The collaboration filtering recommendation system algorithm is first employed to predict circRNA–disease associations. Then, the leave-one-out cross validation mechanism is adopted to measure the performance of our proposed computational method. ICFCDA achieves the areas under the curve of 0.946, which is better than other existing methods. In order to further illustrate the performance of ICFCDA, case studies of some common diseases are made, and the results are confirmed by other databases. The experimental results show that ICFCDA is competent in predicting the circRNA–disease associations.

## Introduction

Circular RNA (circRNA) is a relatively novel biological molecule compared with the usual linear RNAs. Circular RNAs were first discovered in the RNA viruses before 1970 (Sanger et al., 1976). It is said that circRNAs lack covalently closed-loop structures with neither 5′-3′ polarities nor polyadenylated tails (Chen and Yang, 2015), which causes that it is not easy to find circRNAs compared with linear RNAs. Because of circRNAs closed-loop structure, however, more and more circRNAs (Hsu and Coca-Prados, 1979; Arnberg et al., 1980; Pasman et al., 1996) are revealed based on the development of the RNA base sequence high-throughput techniques. In terms of recent researches, there are various kinds of circRNAs in the creatures, which include as follows: exonic circRNAs, which are mainly produced by back-spliced exons (Wilusz and Sharp, 2013), introns circRNAs extracted from introns (Lasda and Parker, 2014), exon–intron circRNAs that are analogous to ecircRNAs (Li et al., 2015), and integrated circRNAs discovered by a biological identifier, CIRI (Gao et al., 2015). Many recent evidences (Danan et al., 2011) show that circRNAs play significant roles in different biological processes and have multiple biological functions (Jeck and Sharpless, 2014; Qu et al., 2015). First, circRNA can be regarded as miRNA sponges (Hansen et al., 2013; Kulcheski et al., 2016), which could be adopted to be an identifier for diseases. Second, some evidences illustrate that circRNAs also can regulate some transcriptional processes (Chao et al., 1998). Simultaneously, circRNAs also have associations with RNA-binding proteins (RBPs) (Panda et al., 2017) bases on their stable circular structures. Circular RNA has different ways to bind with the RBPs compared with the linear RNA (Memczak et al., 2013), which indicates that circRNAs have potential to be disease biomarkers. Moreover, circRNAs have some translational functions (Chen and Sarnow, 1995) like common RNAs.

With the further study of circRNAs’ functions, increasing numbers of evidences point out that circRNAs have associations with complicated diseases (Xu et al., 2017) or have effects on the translation of some proteins (Bartsch et al., 2018). There are many previous searches revealing the associations between circRNAs and some cancers. Circular RNA circ-PVT1 has been discovered to upregulate the gene expression in the gastric cancer (GC) tissues and promotes the GC cells reproduction (Chen et al., 2017a). In contrast circRNA hsa_circ_0000190, it regulates the gene expression in GC tissues by downregulation (Chen et al., 2017b). CircRNA circTCF25 can upregulate the gene expression or cell proliferation of 13 target locus of miRNA miR-103a-3p/miR-107, which can be regarded as a biomarker of bladder cancer (BC) (Zhong et al., 2016). Circular RNA hsa_circRNA_105055 and hsa_circRNA_086376 are the potential biomarkers of colorectal cancer by working as sponges for miR-7 (Zeng et al., 2017). Moreover, circRNA hsa_circ_0054633 also has association with diabetes, especially for prediabetes and type 2 diabetes mellitus (Zhao et al., 2017).

Because of the development of RNA base sequence techniques, more and more circRNA-related information is excavated. Thus, many different kinds of circRNA-related databases are established for further researches of various diseases, biological molecules and pathways, etc. To create more convenience to the researchers, circBase database (Glazar et al., 2014) was developed to provide the evidence supporting their expression, and all the data can be accessed, downloaded, and browsed within the genomic context. Circular RNADb (Chen et al., 2016a) is a comprehensive circRNA database that collects human protein-coding annotations of circRNAs and includes some important information about exonic circRNAs such as genomic information, exon splicing, genome sequence, internal ribosome entry site, open reading frame, and cricRNA-related references. Furthermore, ExoRBase (Li et al., 2017) is an online accessible database that extracts data from RNA-seq data analyses of human blood exosomes. circNet (Lin et al., 2015) is also a circRNA-related database from which tissue-specific circRNA expression profiles and circRNA-miRNA-gene regulatory networks can be downloaded. Moreover, circ2Traits (Ghosal et al., 2013) is an overall circRNA–disease associations database, which obtains the associations as follows: one is identifying the interactions of circRNAs with disease-related miRNAs; the other is matching the diseases associated SNPs on circRNA loci. To obtain more reliable circRNA–disease associations, circR2Disaese (Fan et al., 2018) database (http://bioinfo.snnu.edu.cn/CircR2Disease/) was developed. The whole circRNA–disease associations are collected manually from relevant references and reviews, which provides more convenience and basics to infer novel circRNA–disease associations.

Although, there are many circRNA–disease associations discovered by biological experiments, whose experimental processes are extremely expensive and time-consuming. On the one hand, there are a limited number of computational methods existing to predict potential circRNA–disease associations. On the other hand, we lack comprehensive circRNA-related diseases databases, which are our main motivation to propose a new computational method based on circR2Disease database. In this study, we develop an improved collaboration filtering recommendation system (Pan et al., 2008) method to predict circRNA–disease associations, which is named ICFCDA. First, circRNAs target gene–related gene ontology (GO) terms, circRNAs base corresponding sequences data, and circRNA–disease associations are adopted to calculate the circRNA functional annotation semantic similarity, sequence similarity, and Gaussian interaction profile (GIP) kernel similarity. Second, disease-related genes and circRNA–disease associations are used to calculate the disease functional similarity and disease GIP kernel similarity. Furthermore, we also replace the disease names into disease ontology (DO) IDs to calculate the disease semantic similarity based on the DOSE (Yu et al., 2015) tool. Third, multiple disease similarities and circRNA similarities are combined with the final disease similarity matrix and circRNA similarity matrix, respectively. Finally, collaboration filtering method is adopted to calculate the score of each circRNA–disease pair. For the sake of evaluating the performance of method we proposed, leave-one-out cross validation (LOOCV) is used to calculate the area under receiver operating characteristic (ROC) curve (AUC) value. Moreover, several common diseases also are tested by the LOOCV mechanism. In addition, case studies of two common diseases are implemented to further illustrate the performance of ICFCDA.

## Materials and Methods

### Human circRNA–Disease Associations

To extract circRNA–disease associations, the initial circRNA–disease associations datasets are downloaded from circR2Disease database (Fan et al., 2018) (http://bioinfo.snnu.edu.cn/CircR2Disease/). In the original dataset, there are 725 circRNA–disease associations that have been verified by biological experiments. These 725 circRNA–disease associations contain 661 circRNA individuals and 100 disease individuals. In term of the initial dataset, 212 circRNA–disease associations are picked out as the known associations in this study, which are composed of 42 disease entities and 200 circRNA entities. The adjacency circRNA–disease association matrix is deciphered by matrix *A*. If there is an association between the disease *i* and circRNA *j*, *A*(*i*, *j*) is equal to 1 or *A*(*i*, *j*) is equal to 0.

### circRNA Similarity

#### circRNA Functional Annotation Semantic Similarity

On the basis of the original circRNA–disease associations, 200 circRNA entities are screened out. Then human GO terms data are downloaded from human protein reference database (HPRD) database (Keshava Prasad et al., 2009). The initial circRNA–disease associations provide the circRNAs-related genes. Thus, the circRNA-related genes are utilized to match GO data extracted from HPRD database. In this study, an information content algorithm (Lin, 1998) is adopted to calculate the circRNA functional annotation semantic similarity. *CFS* is used to describe the circRNA functional annotation semantic similarity network. Moreover, the following equation is used to calculate the circRNA functional annotation semantic similarity:

(1) $$\mathrm{CFS}(i,j)=\frac{2\times \log P(C_{i}\cup C_{j})}{\log P(C_{i})+\log P(C_{j})}$$

where *CFS(i, j)* denotes the functional annotation semantic similarity between circRNA *Ci* and *Cj*; *P*(*Ci*) and P(*Cj*) represent the probability between the number of *Ci* and *Cj* target gene–related GO terms and the number of the entire GO terms. $P(C_{i}\cup C_{j})$ is the ratio of between the union of the number of circRNA *Ci* and *Cj* target gene–related GO terms and the number of the entire GO terms.

#### circRNA Sequence Similarity

For the sake of calculating the circRNA sequence similarity, the circRNA corresponding RNA base sequence data are downloaded from circBase database (Glazar et al., 2014) (http://www.circbase.org/). In our computational model, there are 200 circRNAs needing matching their related RNA base sequences. A base pairing algorithm named the Needleman-Wunsch pairwise alignment algorithm is used to calculate the circRNA sequence similarity, which is integrated into a python toolkit called Biopython (Cock et al., 2009). Therefore, there are some parameters needing setting up for obtaining a better result. The gap-open penalty is set as 2, and the gap-open extending penalty is set as −0.5 to −0.1. *CSS* is adopted to represent the circRNA sequence similarity matrix, and *CSS*(*i*, *j*) represents the similarity value between the circRNA *C* *_i_* and *C* *_j_*. Then, the Needleman-Wunsch score of each circRNA pair is normalized as follows:

(2) $$CSS(i,j)=\frac{NW(i,j)}{\sqrt{NW(i,i)}\sqrt{NW(j,j)}}$$

where *NW(i, j)* is the score of the Needleman-Wunsch algorithm between circRNA *i* and *j*.

#### circRNA GIP Kernel Similarity

Known circRNA–disease associations are adopted to calculate circRNA GIP kernel (Van Laarhoven et al., 2011) similarity marked as *CGS*. According to an assumption (Van Laarhoven et al., 2011) that the more similar the two circRNAs are, the more likely the disease associated with one of them is to be associated with another. Therefore, *V* *_Ci_* is used to represent the interaction profile of circRNA *C(i)* with each disease, which means the ith row in the circRNA–disease association network. The GIP kernel similarity between circRNA *C(i)* and *C(j)* is calculated as follows:

(3) $$CGS(i,j)=\exp(-\gamma_{c}{\left\|V_{C_{i}}-V_{C_{j}}\right\|}^{2})$$

where *CGS*(*i*, *j*) is the GIP kernel similarity of circRNA *i* and *j*. γ*_c_* is an adjusting parameter, which controls the bandwidth of each kernel, which can be initialized as follows:

(4) $$\gamma_{c}=\gamma_{c}^{}/\left(\frac{1}{N_{c}}\sum_{i=1}^{N_{c}}\left\|V_{C_{i}}\right\|\right)$$

Where γc^ is the initial value, which is set as 1 based on the previous study (Van Laarhoven et al., 2011). *N* *_c_* is total number of circRNAs.

#### circRNA Similarity Integration

Finally, we obtain the circRNA functional annotation semantic similarity, sequence similarity, and GIP kernel similarity. In order to make full use of these three circRNA similarities, the following equation is adopted to integrate the circRNA similarities:

(5) $$CS(i,j)=\{\begin{array}{c} CGS(i,j),\text{if}CGS(i,j)\neq 0\\ \alpha CFS(i,j)+(1-\alpha )CSS(i,j),\text{otherwise} \end{array}$$

where *CS* denotes the integrated circRNA similarity network; α is a harmonic mean factor to integrate the circRNA functional annotation semantic similarity *CFS*, and the circRNA sequences similarity *CSS*.

### Disease Similarity

#### Disease Functional Similarity

Furthermore, disease-related genes are downloaded from DisGeNET (Pinero et al., 2017) database, which gathers more than 3,815,056 gene–disease associations between 16,666 gene individuals and 13,172 disease individuals. In order to obtain more reliable disease similarity, we also extract disease-related genes from Online Mendelian Inheritance in Man (OMIM) (Hamosh et al., 2005) database. Based on the initial circRNA–disease associations, 42 independent disease entities are picked out as the experimental data. Then, those above disease entities are used to match the disease phenotype corresponding genes in the OMIM dataset manually. In this study, JACCARD algorithm, a statistic method, is used to calculate the disease functional similarity as follows:

(6) $$DS1(i,j)=\frac{\left|DG(i)\cap DG(j)\right|}{\left|DG(i)\cup DG(j)\right|}$$

where *DG(i)* and *DG(j)* denote the subsets of the disease i and j related genes.

#### Disease GIP Kernel Similarity

GIP kernel similarity algorithm is also adopted to calculate the disease GIP kernel similarity between *D*(*i*) and *D*(*j*), which is similar to calculate circRNA GIP kernel similarities. The computing process is as follows:

(7) $$DGS(i,j)=\exp(-\gamma_{d}{\left\|V_{D_{i}}-V_{D_{j}}\right\|}^{2})$$

where *DGS* is the disease GIP kernel similarity network, and the *DGS*(*i*, *j*) is GIP kernel similarity score between disease *i* and *j*. γ*_d_* is also a bandwidth adjustment parameter, which is defined as follows:

(8) γd=γd^/(1Nd∑i=1Nd‖VDi‖)

where γd^ is the initial value, which is set as 1 based on the previous study (Van Laarhoven et al., 2011). *N* *_d_* is total number of diseases.

#### Disease Semantic Similarity

In order to calculate the semantic similarity between these 42 diseases, the disease-relevant DO IDs are extracted from the DO (Kibbe et al., 2015) database. Then all the 42 diseases’ names are replaced into the corresponding DO IDs, which are adopted to input into a R package named DOSE (Yu et al., 2015) to calculate the disease semantic similarity. After the semantic similarity score of each disease pair is obtained, *DS*2 can be used to represent the diseases semantic similarity matrix.

#### Disease Similarity Integration

Thus, the integrated disease similarity thereby can be accessed by combining the disease functional similarity, GIP kernel similarity, and semantic similarity. In this study, when we fuse different disease similarities, different weights are allocated to the disease functional similarity matrix, GIP kernel similarity matrix, and semantic similarity matrix based on the following formula:

(9) $$DS(i,j)=\{\begin{array}{c} DGS(i,j),\text{if}DGS(i,j)\neq 0\\ \beta DS1(i,j)+(1-\beta )DS2(i,j),\text{otherwise} \end{array}$$

where *DS* denotes the integrated disease similarity network.

### ICFCDA

**Figure 1:**
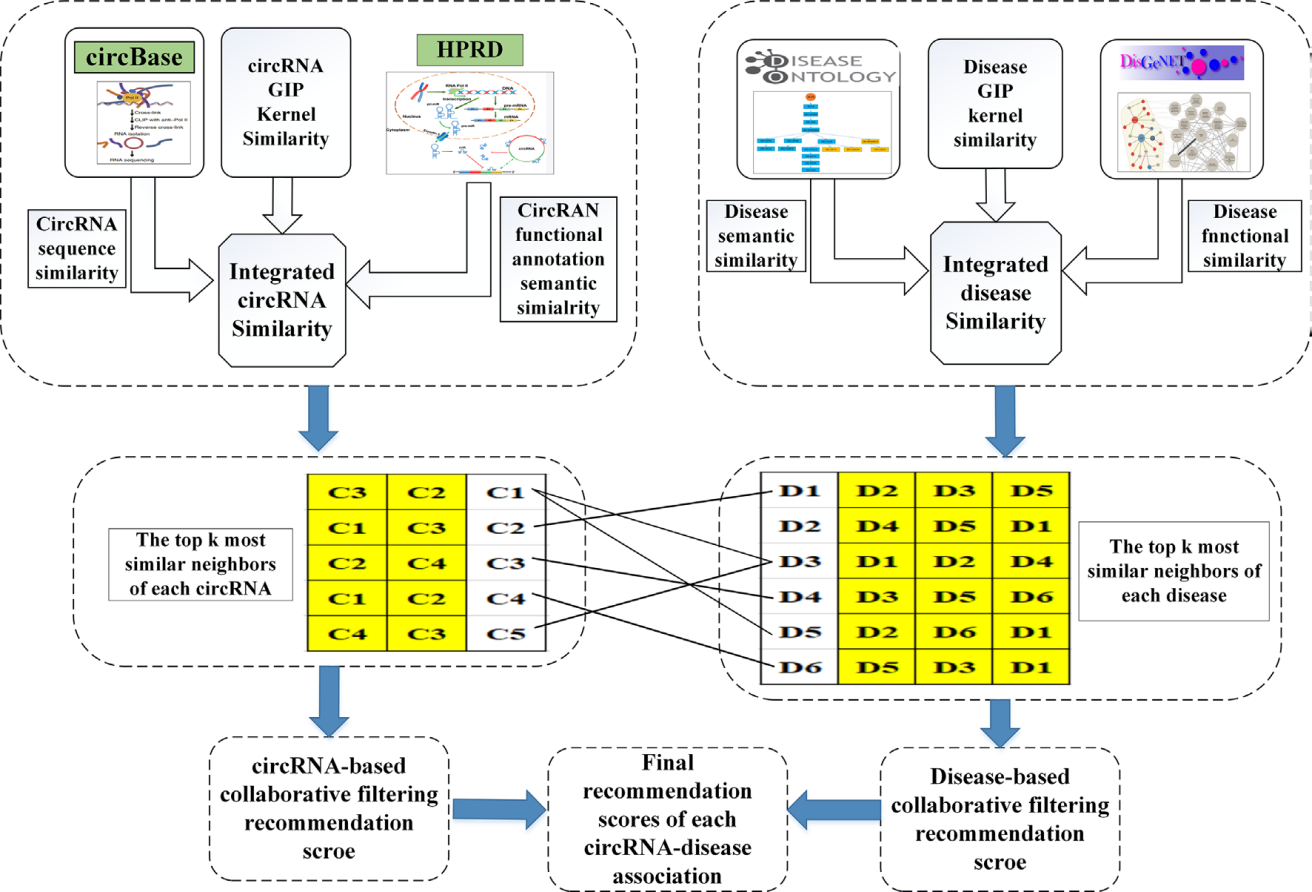
The flowchart of the computational method ICFCDA.

With the increasing numbers of data in all aspects, it is important to predict or recommend some associations between the two different things. It is in this case that the recommendation system algorithm has attracted the attention of many experts. Collaborative filtering algorithm (Schafer et al., 2007; Zhou et al., 2015) is one of the recommendation system algorithms, which is applied to recommend movies (Zhou et al., 2008) or news (Das et al., 2007) for users. In this study, we first adopt the collaborative filtering recommendation system algorithms to predict the circRNA–disease associations, which is named as ICFCDA, and its flowchart is illustrated in **Figure 1**.

For scoring each circRNA–disease association, there are five steps in our computational method as follows:

Step 1: Obtaining the top *k* similar neighbors of each circRNA based on circRNA similarity network *CS*.
Step 2: Obtaining the top *k* similar neighbors of each disease based on disease similarity network *DS*.
Step 3: Calculating the scores of circRNA–disease association by the collaborative filtering recommending based on circRNAs.
Step 4: Calculating the scores of circRNA–disease association by the collaborative filtering recommending based on diseases.
Step 5: Integrating the final recommendation scores based on Steps 3 and 4.

First, the similarity scores between circRNA *j* and other circRNAs in the circRNAs dataset are listed in descending order. Then, the most similar top *k* neighbors of each circRNA are picked out based on the final integrated circRNA similarity network *CS*. We conduct the same above processes for each circRNA. Therefore, we obtain the most similar top *k* neighbors of each circRNA. Furthermore, the value of *k* is set as the 4% of the number of the whole circRNAs, which can be described as nc*0.04.

Second, in terms of the most similar top *k* neighbors of cirRNA *j* and the associations between the disease *i* and the neighbors of the circRNA *j*, the most similar top *k* neighbors of the circRNA-based recommendation score between the disease *i* and the circRNA *j* can be calculated as follows:

(10) $$CRS(i,j)=\frac{1}{k}\left(\sum_{n=1}^{k}A(i,n)\times CS(n,j)\right)$$

where *CRS*(*i*, *j*) is the recommendation score between the disease *i* and the circRNA *j* based on the top *k* most similar neighbors of circRNA *j*. *A*(*i*, *n*) is the association information of the *n*th most similar neighbor of circRNA *j* and the disease *i*. *CS*(*n*, *j*) is the similarity score of the *n*th most similar neighbor circRNA and circRNA *j*.

Third, the similarity scores between disease *i* and other diseases in the disease dataset are listed in descending order. Then, the most similar top k neighbors of each disease are screened out based on the final integrated disease similarity network *DS*. We also carry out the same processes for each disease. Therefore, the most similar top *k* neighbors of each disease are selected. Moreover, the value of *k* is set as the 4% of the number of the whole diseases, which can be described as *nd* * 0.04.

Fourth, based on the most similar top *k* neighbors of disease *i* and the associations between the neighbors of the disease *i* and the circRNA *j*, the most similar top *k* neighbors of the disease-based recommendation score between the disease *i* and the circRNA *j* can be calculated as follows:

(11) $$DRS(i,j)=\frac{1}{k}\left(\sum_{m=1}^{k}DS(i,m)\times A(m,j)\right)$$

where *DRS*(*i*, *j*) is the recommendation score between the disease *i* and the circRNA *j* based on the top *k* most similar neighbors of disease *i*. *A*(*m*, *j*) is the association information of the *m*th most similar neighbor of disease *i* and the circRNA *j*. *DS*(*i*, *m*) is the similarity score of the *m*th most similar neighbor disease and disease *i*.

Finally, the circRNA-based recommendation scores and the disease-based recommendation scores are combined with the final recommendation scores as follows:

(12) IRS(i,j)=γDRS(i,j)+(1−γ)CRS(i,j)

where *IRS*(*i*, *j*) is the integrated recommendation scores between the disease *i* and the circRNA *j*. The parameter γ∈[0, 1.0] is a balance factor that can control the significance of the circRNA-based recommendation scores and the disease-based recommendation scores.

In order to solve the “cold start” problem in the collaborative filtering recommendation system, the importance of neighbors is taken into consideration. The more diseases/circRNAs are shared by two cicRNAs/diseases, the more significant it is. The importance of two diseases/circRNAs can be defined as follows:

(13) $$IMP(C(i),C(j))=f_{exp}(C(i))*f_{ns}(C(j))*\sum_{C(c(k))}f_{cod}(c(k))$$

where *IMP*(*C*(*i*), *C*(*j*)) is the significance coefficient between circRNA *i* and *j*. *IMP* is divided into three parts, which include the circRNA *C*(*i*) related diseases *f* *_exp_*(*C*(*i)*), which can be calculated as the following equation:

(14) $$f_{exp}(C(j))=\frac{1}{D(C(i))}$$

where *D*(*C*(*i*)) is circRNA *i–*related diseases, which means that circRNA *i* would provide more useful suggestion, if it is associated with fewer diseases. *f* *_ns_*(*C*(*j*)) is the similarity if disease *j *based on the disease *i*, which is defined as follows:

(15) $$f_{ns}(C(j))=\frac{1}{D(C(j))-I(C(i),C(j))+1}$$

where *I*(*C*(*i*), *C*(*j*)) is intersection of the circRNA *i* and *j*–related disease dataset. *f* *_cod_*(*C*(*k*)) is the disease that is merely associated with circRNA *i* and *j*. Therefore, for those circRNAs that have only one relevant disease, the following equation is adopted to calculate the recommendation score:

(16) $$Score_{cold\;start}=\sum_{i=1}^{Nc}IMP(C(t),C(i))*CS(C(t),C(i))$$

### Performance Metric

In order to evaluate the performance of our proposed computational method, the AUC value that is the area of the ROC curve and the *f*-measure, which is a comprehensive metric using the *precision* and the *recall*, are the two main evaluation metrics in this study. The ROC curve consists of the true-positive rate (TPR) and the false-positive rate (FPR), which are calculated by the following equations:

(17) $$TPR=\frac{TP}{TP+FN}$$

(18) $$FPR=\frac{FP}{FP+TN}$$

where TP is the number of the positive samples that is the known circRNA–disease associations, which are predicted as the true circRNA–disease associations, and FN is the number of the negative samples predicted as the false circRNA–disease associations. FP is the number of the incorrectly predicted positive samples, and the TN is the number of the truly predicted negative samples. In addition, the *precision* is the true-positive samples in the dataset, which are predicted as positive samples dataset. The *recall* is the ratio between the samples that are predicted as positive samples and the whole positive samples. Thus, *f*-measure is illustrated as follows:

(19) $$precision=\frac{TP}{TP+FP}$$

(20) $$recall=\frac{TP}{TP+FN}$$

(21) $$f-measure=\frac{2\times precision\times recall}{precision+recall}$$

## Results

### Leave-One-Out Cross Validation

**Figure 2:**
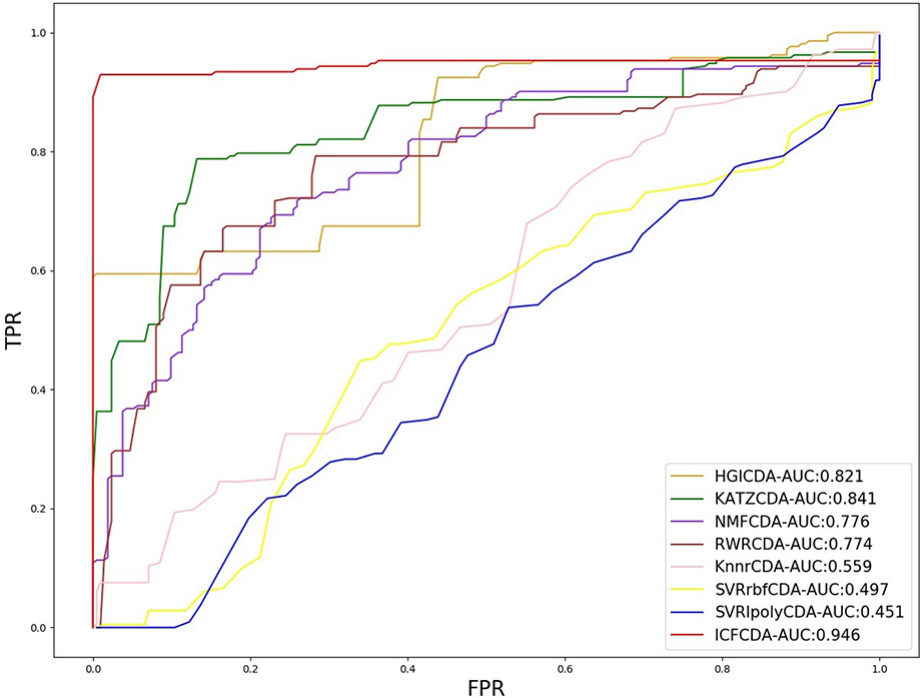
The AUC value of ICFCDA compared with other computational methods.

**Figure 3:**
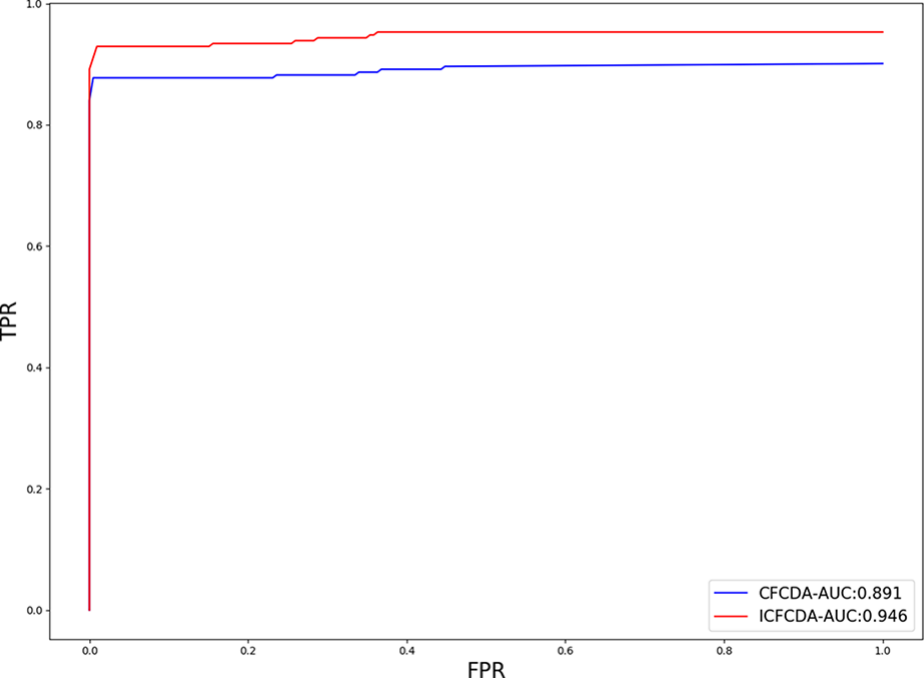
The AUC value of ICFCDA compared with CFCDA without solving the “cold start” problem.

**Figure 4:**
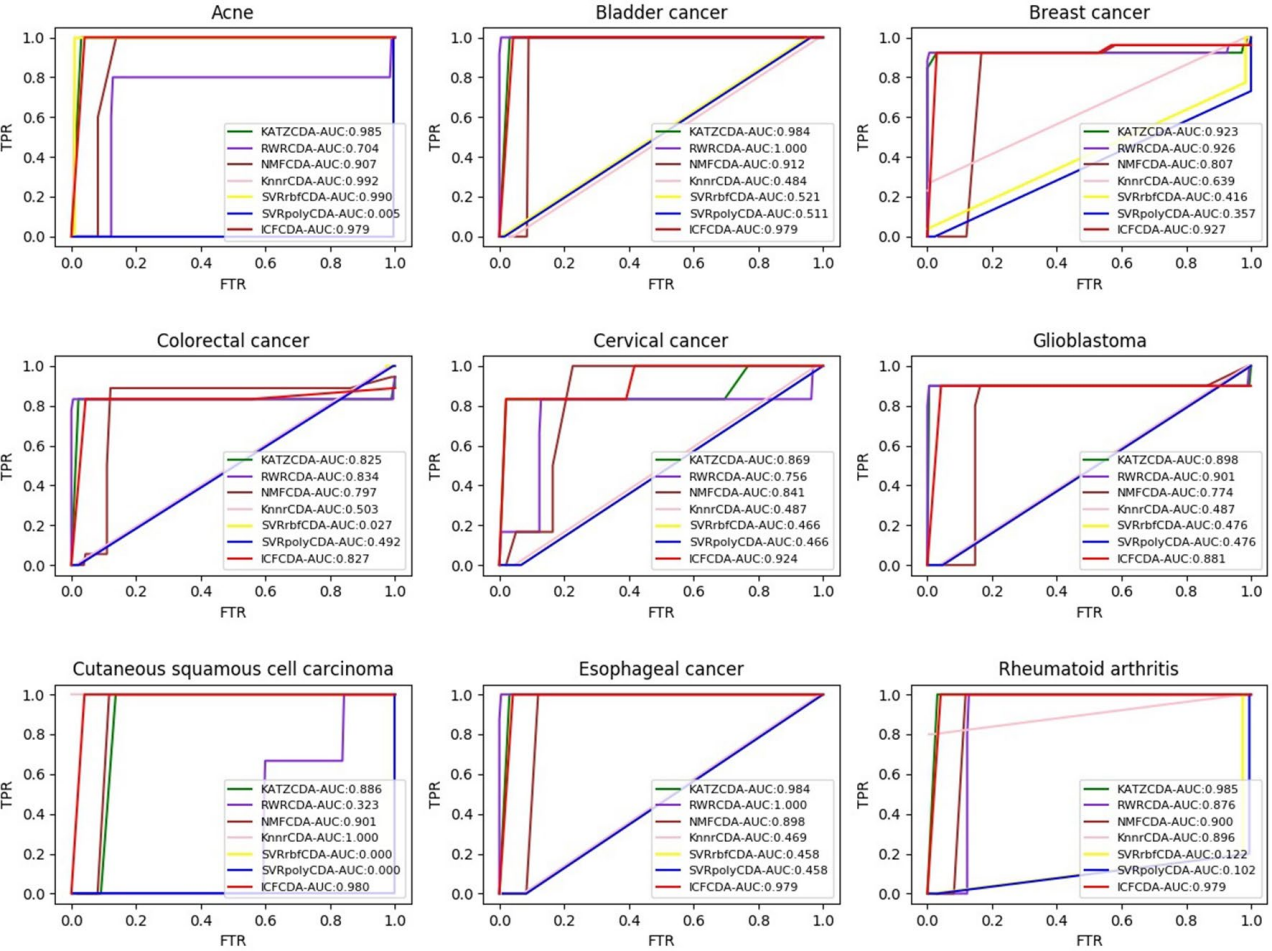
The AUC values of nine kinds of specific diseases.

**Figure 5:**
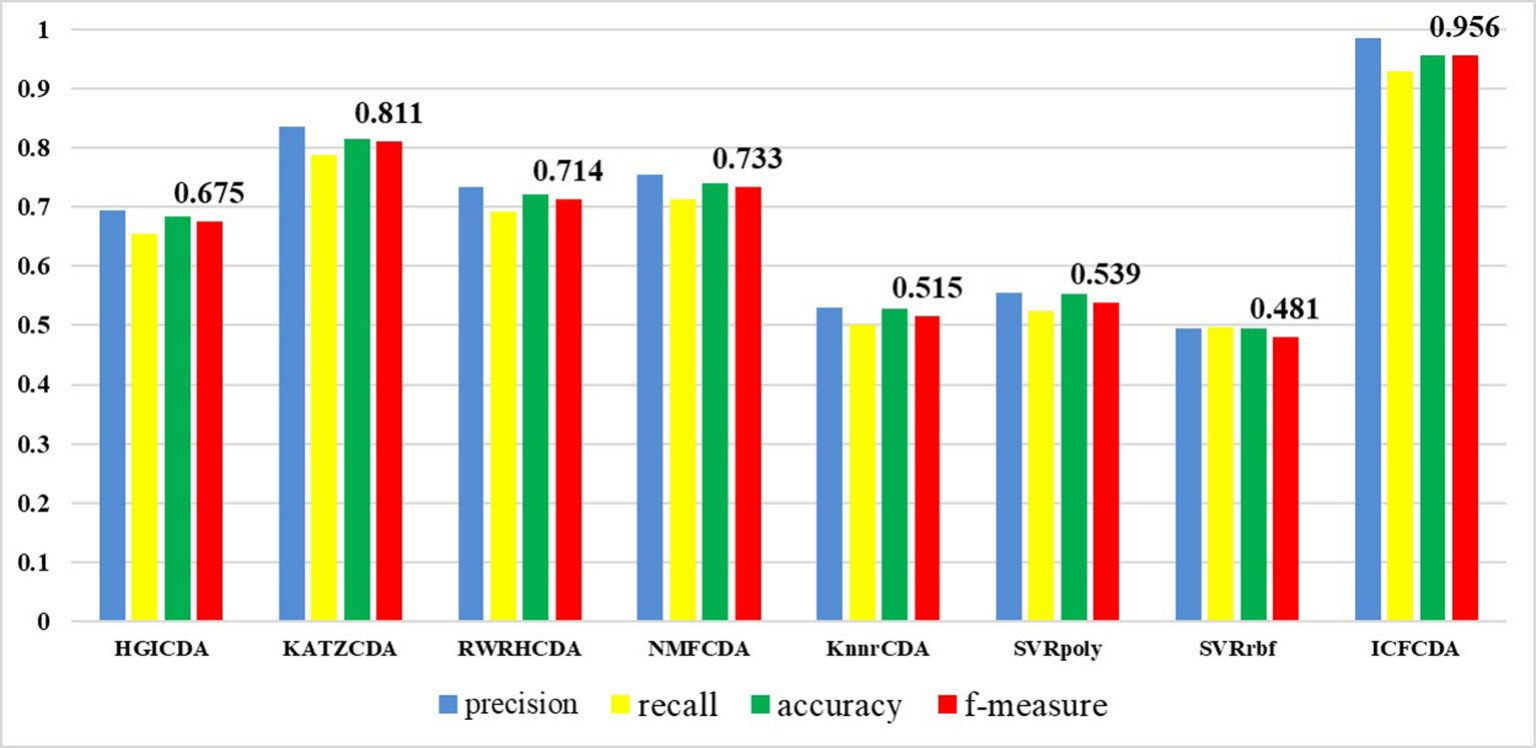
Comparison of the precision, recall, accuracy, and *f*-measure with different methods.

**Figure 6:**
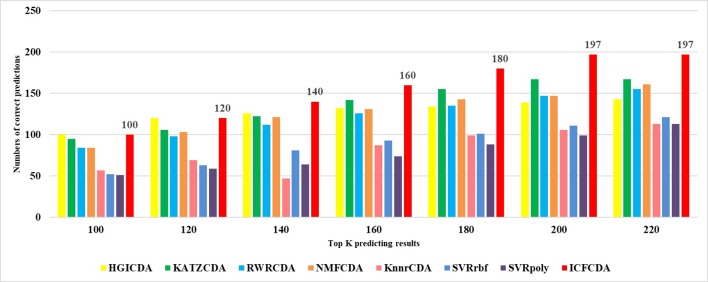
The number of correct circRNA–disease association in top *k* predicting results.

In this study, a cross validation mechanism, LOOCV, is adopted to test the performance of our proposed computational method, ICFCDA. For each given disease in the circRNA–disease association network, there could be one or several relevant circRNAs with each specific disease. First, for each given disease *i*, some circRNAs are confirmed that they are associated with the disease *i*, which are the known circRNA–disease associations. Each association between the disease *i* and one particular circRNA could be regarded as test data, and all the left circRNA–disease associations are seen as training dataset. During each LOOCV procedure, one circRNA–disease association potential score is generated. When all the scores of the test dataset are obtained, the remaining unknown circRNA–disease associations are treated as the test dataset. Finally, the predictive score of each circRNA–disease pair is obtained. Each circRNA–disease association score is a threshold after the final potential scores of the circRNA–disease associations are sorted in descending order. With the changing threshold, we can calculate the TPRs and the FPRs, which are adopted to draw the ROC curve and calculate the AUC value. In order to evaluate the performance of ICFCDA, the AUC value is compared with other seven state-of-the-art methods such as heterogeneous graph inference (HGI) method (Chen et al., 2016b), KATZ (Ganegoda et al., 2014), random walk restart (RWR) (Chen et al., 2012), and graph regularized nonnegative matrix factorization (NMF) (Liu et al., 2018), respectively. The result is shown in **Figure 2**, which illustrates that the performance of ICFCDA is better than others. According to **Figure 2**, we can find that ICFCDA achieves greater AUC value of 0.946 compared with HGI (0.821), KATZ (0.841), RWR (0.774), NMF (0.776), K-nearest neighbor regression (0.559), support vector regression with rbf kernel (0.497), and support vector regression with poly kernel (0.451), respectively. Moreover, the experiment of collaborative filtering without solving the “cold start” problem is supplemented to evaluate the performance of ICFCDA, which is presented in **Figure 3**. We also make the prediction of other nine common diseases including BC, breast cancer, colorectal cancer, and so on, which are represented in **Figure 4**. In order to illustrate the stability of our proposed computational method, the average AUC values based on the 42 diseases of other methods are shown in **Table 1**. Based on **Figure 2** and **Table 1**, ICFCDA can obtain better and more stable performance than other computational methods. Furthermore, for the sake of obtaining more comprehensive and reliable results, *f*-measure is also treated as one of our evaluating metric, which is described in **Figure 5**. In addition, we also show the first *k* correct circRNA–disease relationships in the predicting results, which is described in **Figure 6**.

**Table 1:** The average AUC values of 42 diseases.

	KATZCDA	RWRCDA	NMFCDA	KNNR	SVRrbf	SVRpoly	ICFCDA
Average AUC	0.719	0.478	0.616	0.536	0.441	0.415	0.885

### Parameters Analysis

**Figure 7:**
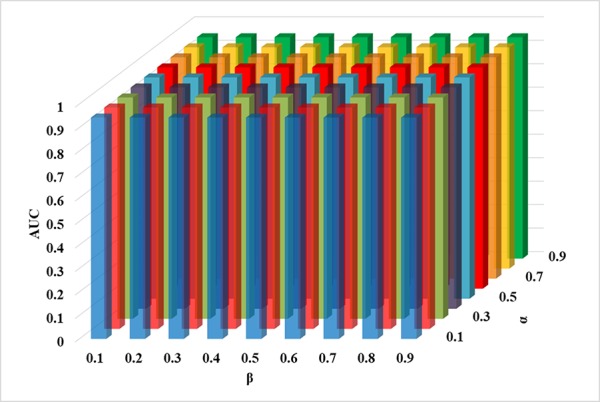
The AUC of the parameter α and β based on the fixed parameter γ and *k*.

In this study, there are three main parameters that are the most similar top *k* neighbors of each circRNA/disease, the circRNA similarity integration adjustment factor α and the disease similarity integration adjustment factor β, respectively. Parameter *k* controls the selecting neighbors’ number of each circRNA/disease, which provides the recommendation information from neighbors. The parameter α determines the importance between the circRNA functional annotation semantic similarity and the circRNA sequence similarity, and its value is changed from 0.1 to 0.9. The third parameter β is a tradeoff between the disease functional similarity and the disease semantic similarity, whose value ranges from 0.1 to 0.9. At first, to avoid causing the bias between the circRNA and the disease recommendation scores, the recommendation integration factor γ is set as *N* *_c_*/(*N* *_d_*+*N* *_c_*), where *N* *_c_* is the number the circRNA entries, and the *N* *_d_* is the number of the disease entries. At first, for testing the suitable value of the parameter *k*, the parameter α and the parameter β and γ are set up as 0.5, 0.5, and *N* *_c_*/(*N* *_d_*+*N* *_c_*), which means that different disease similarity scores are treated equally. According to the above experiments, the parameter α, β, and γ are fixed. When *k* is set as 4%, ICFCDA can obtain the best AUC value (0.946), which is shown in **Table 2**. After that, we can find that the parameter α and β are not sensitive in our computational method according to **Figure 7**. Therefore, both the parameter α and β are set as 0.5.

**Table 2:** AUC with different values for parameter *k*.

k	1	2	3	4	5	6	7	8	9	10
AUC	0.930	0.932	0.940	**0.946**	0.923	0.921	0.921	0.906	0.906	0.902

### Case Study

In order to further evaluate the performance of our proposed computational method ICFCDA, we also conduct case studies of two common diseases in the world, which are BC (Kaufman et al., 2009) and breast cancer (Veronesi et al., 2005). Bladder cancer is one of the most common genitourinary malignant diseases, which has caused hundreds of thousands of people’s death since it was discovered clinically. What’s worse, the risk of BC increases with the increasing age. Another case study is about the breast cancer, which is an important public healthy disease worldwide and is also hard to prevent. Breast cancer has a very high mortality rate. Therefore, some computational methods should be put forward to identify the potential biomarkers of these above two diseases. In this study, the prediction results of ICFCDA are validated by the other three circRNA–disease association–related databases, which are the circ2Disease (Yao et al., 2018), circRNADisease (Zhao et al., 2018), and LncRNADisease v2.0 (Bao et al., 2019), which are shown in **Tables 3** and **4**. Both **Tables 3** and **4** are the predicting results of the top 10 BC- and breast cancer–relevant circRNAs. Circ2Disease, circRNADiseaes, and LncRNADisease are represented by *, #, and +, respectively.

**Table 3:** The top 10 bladder cancer related candidates’ circRNAs.

Rank	CirRNA name/id	Evidences	Rank	CircRNA name/id	Evidences
**1**	hsa_circ_0000172	+	**6**	hsa_circ_0002024	+
**2**	hsa_circ_0002495	+	**7**	circMylk/ circRNAMYLK/ hsa_circ_0002768	*, #
**3**	circRNABCRC4/ hsa_circ_001598/ hsa_circ_0001577	PMID: 29270748	**8**	circTCF25/ hsa_circ_0041103	#
**4**	hsa_circ_0003221/ circPTK2	#, +	**9**	circFAM169A/ hsa_circ_0007158	#
**5**	hsa_circ_0091017	#, +	**10**	circTRIM24/ hsa_circ_0082582	#

**Table 4:** The top 10 breast cancer–related candidates’ circRNAs.

Rank	CirRNA name/id	Evidences	Rank	CircRNA name/id	Evidences
**1**	hsa_circ_0011946	+	**6**	circAmotl1/ hsa_circ_0004214	*, #
**2**	hsa_circ_0093859	+	**7**	hsa_circ_0006528	*, #, +
**3**	hsa_circ_0001982	#, +	**8**	hsa_circ_0002874	#, +
**4**	hsa_circ_0001785	#, +	**9**	hsa_circ_0085495	#, +
**5**	hsa_circ_0108942	#, +	**10**	hsa_circ_0086241	#, +

## Conclusion

With the discovery of an increasing numbers of disease-related circRNAs, more and more attention is paid by biologists. People might have lots of interests to explore the complicated associations between the various kinds of diseases and circRNAs. Simultaneously, because of the development of the RNA high-throughput techniques, it makes more convenience to find the potential associations of circRNAs and diseases. While the RNA high-throughput techniques can make this procedure easier than before, it is not only time consuming but also expensive, which becomes the main motivation to develop a computational method to predict the circRNA–disease associations. In this study, we propose a collaborative filtering recommendation system solving the “cold start” problem-based method to predict the circRNA–disease associations, which is named ICFCDA. For evaluating the performance of ICFCDA, LOOCV and *f*-measure show that ICFCDA can obtain better results than other novel computational methods. Moreover, case studies of BC and breast cancer also are adopted to test the performance of the ICFCDA. In terms of the different evaluations, we believe that our proposed computational method is a useful method to predict the associations of the circRNAs and the diseases.

ICFCDA can obtain better performance because of some following nonnegligible reasons. First, our proposed computational method is based on the recommendation system algorithm, collaborative filtering, which is suitable to be used to predict the circRNA–disease associations. Because circRNAs can be treated as the items, and the diseases can be regarded as the users, the different items (circRNAs) can be recommended to different users (diseases). Second, in order to solve the “cold start” problem, the circRNA similarity and the disease similarity are involved to figure out this problem. For obtaining more reliable recommendation information, various kinds of biological data are adopted to measure the circRNA and disease similarity. We download the circRNA-related gene annotation terms to calculate the circRNA functional annotation semantic similarity and the RNA base sequences to calculate the circRNA sequence similarity. Disease-related genes and phenotypes (DO ID) are used to calculate the disease functional and semantic similarity, respectively. Third, in order to screen out more informative information from the noise, we merely use the top 4% most similar neighbors of each circRNA and disease to obtain more reliable recommendation score.

For the future work, more biological data will be added to calculate the disease and the circRNA similarity for reducing the useless noisy information. Adding multiple data can enrich the information of the different biological network, such as circRNA-lncRNA, circRNA-miRNA, and so on.

## Data Availability

Publicly available datasets were analyzed in this study. This data can be found here: http://bioinfo.snnu.edu.cn/CircR2Disease/article/DownLoad.aspx, http://www.circbase.org/cgi-bin/downloads.cgi, http://www.disgenet.org/downloads, http://www.disease-ontology.org/, http://hprd.org/, https://www.omim.org/.

## Author Contributions

XL conceptualized the algorithm, designed the method, and drafted the manuscript. ZF designed the method and drafted the manuscript. ZF and LG analyzed the data and carried out the experiments. XL modified the manuscript and polished the English expression.

## Funding

This work was supported by the funding from National Natural Science Foundation of China (61972451, 61672334, 61902230) and the Fundamental Research Funds for the Central Universities (No. GK201901010).

## Conflict of Interest Statement

The authors declare that the research was conducted in the absence of any commercial or financial relationships that could be construed as a potential conflict of interest.
